# Ordinal vs dichotomous analyses of modified Rankin Scale, 5-year outcome, and cost of stroke

**DOI:** 10.1212/WNL.0000000000006554

**Published:** 2018-11-20

**Authors:** Aravind Ganesh, Ramon Luengo-Fernandez, Rose M. Wharton, Peter M. Rothwell

**Affiliations:** From the Centre for Prevention of Stroke and Dementia, Nuffield Department of Clinical Neurosciences, University of Oxford, UK.

## Abstract

**Objective:**

To compare how 3 common representations (ordinal vs dichotomized as 0–1/2–6 or 0–2/3–6) of the modified Rankin Scale (mRS)—a commonly used trial outcome measure—relate to long-term outcomes, and quantify trial ineligibility rates based on premorbid mRS.

**Methods:**

In consecutive patients with ischemic stroke in a population-based, prospective, cohort study (Oxford Vascular Study; 2002–2014), we related 3-month mRS to 1-year and 5-year disability and death (logistic regressions), and health/social care costs (generalized linear model), adjusted for age/sex, and compared goodness-of-fit values (C statistic, mean absolute error). We also calculated the proportion of patients in whom premorbid mRS score >1 or >2 would result in exclusion from trials using dichotomous analysis.

**Results:**

Among 1,607 patients, the ordinal mRS was more strongly related to 5-year mortality than both the 0–1/2–6 and 0–2/3–6 dichotomies (all *p* < 0.0001). Results were similar for 5-year disability, and 5-year care costs were also best captured by the ordinal model (change in mean absolute error vs age/sex: −$3,059 for ordinal, −$2,805 for 0–2/3–6, −$1,647 for 0–1/2–6). Two hundred forty-four (17.1%) 3-month survivors had premorbid mRS score >2 and 434 (30.5%) had mRS score >1; both proportions increased with female sex, socioeconomic deprivation, and age (all *p* < 0.0001).

**Conclusion:**

The ordinal form of the 3-month mRS relates better to long-term outcomes and costs in survivors of ischemic stroke than either dichotomy. This finding favors using ordinal approaches in trials analyzing the mRS. Exclusion of patients with higher premorbid disability by use of dichotomous primary outcomes will also result in unrepresentative samples.

The modified Rankin Scale (mRS) has been widely used as an outcome measure in stroke trials^1^ and in more than 30 other neurologic conditions including Parkinson disease and autoimmune encephalitis (data available from Dryad, table e-1, doi.org/10.5061/dryad.609bp7m). However, there is lack of consensus on how the mRS should be analyzed, with more than half of stroke trials since 2007 still using dichotomous analysis, splitting the mRS into favorable/unfavorable outcomes, while about a quarter used ordinal (shift) analysis, assessing all changes across the range.^2^ There is also disagreement about optimal dichotomy, with many recent trials using 0–1/2–6 instead of 0–2/3–6.^3,4^

Dichotomous analyses provide results that are easily explained using the absolute risk reduction in outcome between treatment groups but they may often be statistically inefficient, requiring larger sample sizes, and risk overvaluing a beneficial transition at one end of the distribution while disregarding harm at the other.^5^ The ordinal approach, first advocated for stroke trials by Bath et al.^6^ and Saver,^7^ reduces this risk and allows trials to reduce sample size by 14% to 53%^8^—particularly appealing for trials in rarer conditions—but might also reduce statistical power if benefit is clustered at a few predictable state transitions.^9^ When seeking a clinically interpretable parameter (common odds ratio), ordinal analysis is also limited by the proportional odds assumption, although alternatives have been proposed.^10^ The dilemma is complicated by interrater variability, which is greatest for grades 1 and 2, around which the mRS is dichotomized.^11^

Aside from the issue of which approach is most statistically robust for short-term outcome analysis, the long-term trajectories of patients in different mRS grades can also inform the choice of primary outcome analysis, by testing the validity of the ordinal vs dichotomous forms of the mRS as they relate to long-term clinical or health-economic outcomes.^12^ If meaningful differences in outcomes are seen across the range of mRS scores, resulting in a significantly better fit in regression models using the ordinal mRS, this would favor ordinal approaches, but if meaningful differences are seen only between certain groupings of mRS levels (e.g., mRS 0–2 vs 3–6), this would favor dichotomization.

Moreover, a dichotomous approach usually results in exclusion of patients with premorbid disability, since patients with mRS score >1 or >2 before their stroke cannot achieve a “favorable” poststroke outcome if defined as a 3-month mRS of 0–1 or 0–2, respectively. For example, recent trials of thrombectomy in acute stroke generally excluded patients with premorbid mRS score ≥2.^13^ However, such patients could still contribute in ordinal analysis. It is unclear to what extent dichotomies risk excluding key segments of the population, potentially limiting the external validity of results; quantifying this risk may help trialists decide whether to dichotomize the mRS.

We therefore compared how the 3 commonly used mRS representations (ordinal/full-range vs dichotomized as 0–1/2–6 or 0–2/3–6) relate to 5-year disability, survival, and health/social care costs in 3-month survivors of ischemic stroke in a population-based study. We also examined the proportion of patients who had premorbid mRS score >1 or >2—who would risk exclusion with dichotomous approaches—and examined whether the relationship of the mRS representations to long-term outcomes would differ on excluding these patients.

## Methods

The Oxford Vascular Study population comprises all individuals registered with about 100 general practitioners (GPs) in 9 practices across Oxfordshire, UK (midpoint population between 2002 and 2012 was 92,728; corresponding estimated midpoint population in Oxfordshire was 639,900).^14^ The published characteristics of the study population, such as age and sex structure, closely resemble those of the general population of the United Kingdom.^14^ Recruitment began in April 2002 and is ongoing. Near-complete ascertainment^15^ of suspected stroke or TIA cases is achieved using multiple overlapping methods of “hot” and “cold” pursuit: (1) a daily, rapid access clinic to which participating GPs and the local emergency department refer all individuals with suspected TIA/stroke but are not hospitalized; (2) daily searches of admissions to medical, cardiology, stroke, neurology, and other relevant wards; (3) daily searches of the local emergency attendance register; (4) daily searches of in-hospital death records via the Bereavement Office; (5) monthly searches of all death certificates and coroners' reports for out-of-hospital deaths; (6) monthly searches of GP diagnostic coding and hospital discharge codes; and (7) monthly searches of all brain and vascular imaging referrals.

Consenting patients with ischemic stroke recruited from April 2002 to March 2014 were included. Patients were assessed urgently by study clinicians and considered for inclusion. Stroke was diagnosed per the World Health Organization definition.^16^ Assessments of neurologic impairment, clinical presentation, medical/social history, and risk factors were made. All cases were reviewed by a senior neurologist (P.M.R.) daily, and imaging results were assessed by the study neuroradiologist. Patients were followed up face to face by a study nurse or physician either in a hospital clinic or at home at 1, 3, and 6 months, 1 year, and 5 and 10 years. Recurrent vascular events and disability (mRS) were recorded at each follow-up. Raters were all trained in the use of the mRS using an instructional DVD with accompanying written materials produced by the University of Glasgow that has been used in large-scale clinical trials.^17^

Patients who moved out of the study area were followed up by telephone. Additional information was obtained from a carer in patients with impaired cognition or speech. All deaths were recorded via death certificates, coroners' reports, and the Office for National Statistics Central Register. Health and social care resource use was obtained from the date of the first stroke in the study period (“index” stroke) until 5 years poststroke or May 15, 2017, whichever was first. The methods on how resource use and costs were collected have been reported previously.^18,19^ Briefly, patients' medical records from the Oxford University Hospitals Trust were reviewed for any emergency visit/transport, outpatient-care visit, day case, or hospitalization. For each stay in hospital, the dates of admission, discharge, and transfers between specialty wards were recorded. We estimated the number of institutionalized days as the difference between either date of 5-year follow-up or death, whichever was earliest, and the date of admission into the institution. Hospital resource use was valued using unit costs from the National Health Service's schedule of reference costs.^20^ Institutionalization cost was recorded as the cost per week in a private nursing home, £795 ($1,145) in 2016.^21^ All costs were presented in 2016 prices and converted from UK pounds sterling (£) to US dollars ($) using the 2016 rate of purchasing power parities ($ = £0.694, stats.oecd.org/).

### Standard protocol approvals, registrations, and patient consents

The study was approved by the Oxfordshire Research Ethics Committee. Informed consent was obtained from patients whenever possible; otherwise, assent was obtained from caregivers if patients were unable to consent.

### Statistical analyses

Since we sought to relate the 3-month mRS (as used in trials) to 5-year clinical outcomes and costs, and 3-month mRS = 6 (death) would be perfectly correlated with 5-year death/disability, we focused on patients surviving 3 months past their index stroke. However, since 3-month deaths would contribute to the primary outcome analysis of a trial and might improve the associations of each mRS representation with long-term outcomes to different extents, we ran regressions for 1- and 5-year death both including and excluding these patients.

The proportions of 3-month survivors who had premorbid mRS score >2 or >1 were calculated both for the overall cohort and in relation to age (<45, 45–54, 55–64, 65–74, 75–84, ≥85 years), sex, and index of socioeconomic deprivation. The proportions disabled, dead/disabled, or dead at 1 year and 5 years were also calculated and stratified by 3-month mRS. Disability at 5 years was defined as mRS score >2, but analysis was repeated using mRS score >1. Logistic regression was used to adjust associations of 3-month mRS and long-term outcomes for age/sex. Proportions of interest were compared using χ^2^ tests.

Analyses were censored at May 15, 2017. Since we did not yet have full 5-year data for patients recruited after May 15, 2012, we examined the effect of censoring on costs,^22^ partitioning the study period into smaller time periods (by day) within each of which the total cost incurred for all patients alive at the beginning of the period was calculated. Estimated costs of patients with complete data for each time period were weighted by the Kaplan-Meier sample average estimator and summed over all periods to estimate mean censor-adjusted costs. Costs were stratified by 3-month mRS and reported as means with 95% confidence intervals from 1,000-bootstrap estimates. To assess whether health/social care costs varied over time by 3-month mRS, we constructed generalized gamma linear models (GLMs) assuming a log identity, adjusted for age and sex. Statistical significance was set at *p* < 0.050.

We compared how each mRS representation related to disability and/or death using the area under the receiver operating characteristic (ROC) curve (AUC or C statistic) from age- and sex-adjusted logistic regressions, with each of the following forms of the 3-month mRS: (1) dichotomized as 0–2/3–6, (2) dichotomized as 0–1/2–6, and (3) including the full range of the mRS in the models. Therefore, the 3-month mRS was modeled in logistic regressions as either a binary variable (0–1 vs 2–5 or 0–2 vs 3–5) or a categorical variable with 6 levels (0, 1, 2, 3, 4, 5). Given that increases in goodness of fit in models using the ordinal mRS may relate to the increased number of parameters estimated, we used k-fold cross-validation to generate the AUCs for each logistic regression model (used for disability, death/disability, or death). This involves randomly splitting the dataset into k equally sized groups (we used k = 10). Data on one group is excluded, and the model is fitted to the data on the other k − 1 groups. The resulting model is then applied to the excluded group, and the AUC is calculated. This process is repeated k times, excluding each of the groups in turn. The resulting k AUCs are averaged to produce a single, overall, optimism-corrected estimate of the AUC.^23^ We compared these AUCs using the standard nonparametric approach and calculated the ∆AUC by subtracting the AUC obtained from a logistic model including only age and sex.^24^

We compared the goodness of fit for each mRS representation as it related to 5-year health and social care costs using k-fold cross-validation to estimate the mean absolute error (MAE) for age- and sex-adjusted GLMs containing each representation. We also calculated the ∆MAE vs a GLM including only age/sex (more negative values indicating better goodness of fit).

These analyses were repeated after excluding patients with premorbid mRS score >2, then premorbid mRS score >1. Analyses were performed using Stata 13.1 software (StataCorp LP, College Station, TX).

### Data availability

Requests for access to the data used in this report will be considered by the corresponding author.

## Results

**Figure 1:**
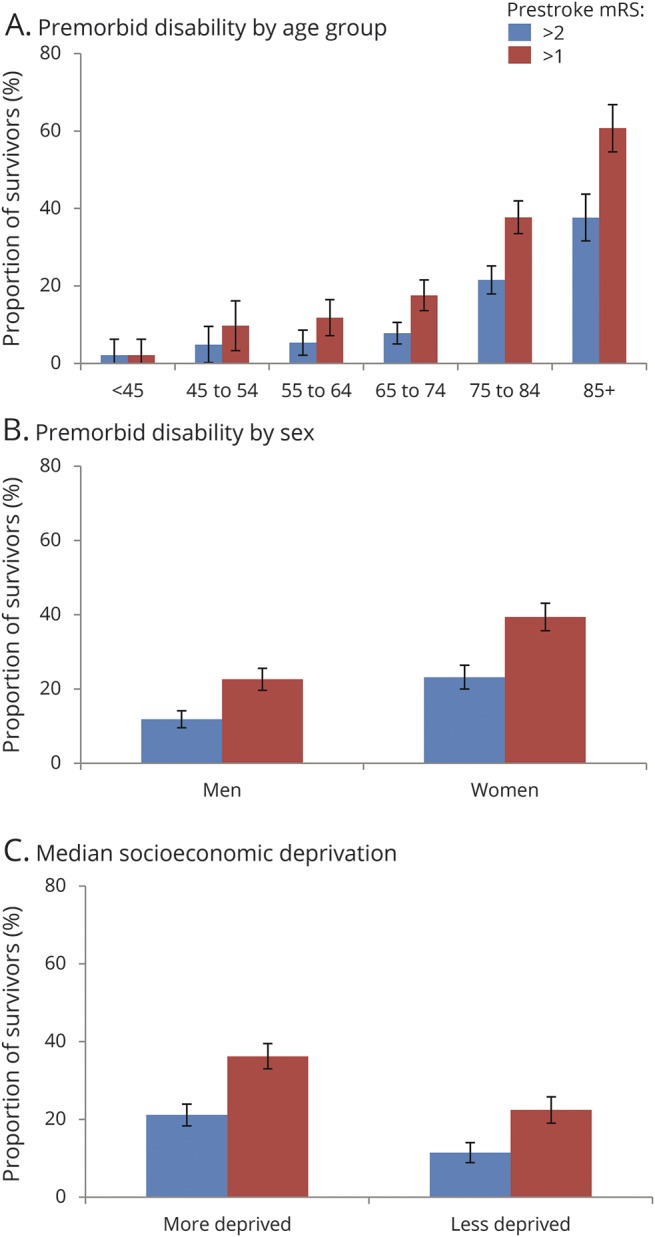
Proportion of 3-month survivors of ischemic stroke with premorbid disability Premorbid disability was defined as a prestroke modified Rankin Scale (mRS) score of >2 (blue) or >1 (red) and is presented by (A) age, (B) sex, and (C) socioeconomic deprivation index. Bars represent 95% confidence intervals.

Of 1,607 patients with an index ischemic stroke between 2002 and 2014, 181 (11.3%) died within 3 months. Complete baseline data were available for 1,421 (99.6%) 3-month survivors (table 1) and follow-up data for 1,403 (98.4%) survivors. Of the 23 excluded survivors, 19 refused inclusion and follow-up (refusal rate of 1.3%) and 4 had mRS assessments only beyond 3 months. Two hundred forty-three (17.1%) 3-month survivors had a premorbid mRS score >2; this proportion increased with age from 5 patients (3.9%) younger than 55 years, to 228 (20.6%) older than 65 years, and 92 (37.9%) older than 85 years (figure 1, A). Four hundred thirty-three patients (30.5%) had a premorbid mRS score >1, including 9 patients (7.0%) younger than 55 years, 402 (36.3%) older than 65, and 150 (60.7%) older than 85. Women were more likely than men to have a premorbid mRS score >2 (155/670 [23.1%] vs 89/752 [11.8%]) or >1 (264/670 [39.4%] vs 170/752 [22.6%], *p* < 0.0001; figure 1, B). Patients at or worse than the median socioeconomic deprivation index were more likely than those less deprived to have a premorbid mRS score >2 (175/828 [21.1%] vs 68/593 [11.5%]) or >1 (300/828 [36.2%] vs 133/593 [22.5%], *p* < 0.0001; figure 1, C). Even when considering only the 1,268 3-month survivors with first-in-lifetime strokes, 188 (14.8%) had a premorbid mRS score >2 and 347 (27.4%) had a premorbid mRS score >1. Age, female sex, and deprivation independently predicted premorbid disability on multivariable logistic regression even upon adjusting for significant comorbidities, including prior stroke (data available from Dryad, table e-2, doi.org/10.5061/dryad.609bp7m).

**Table 1:**
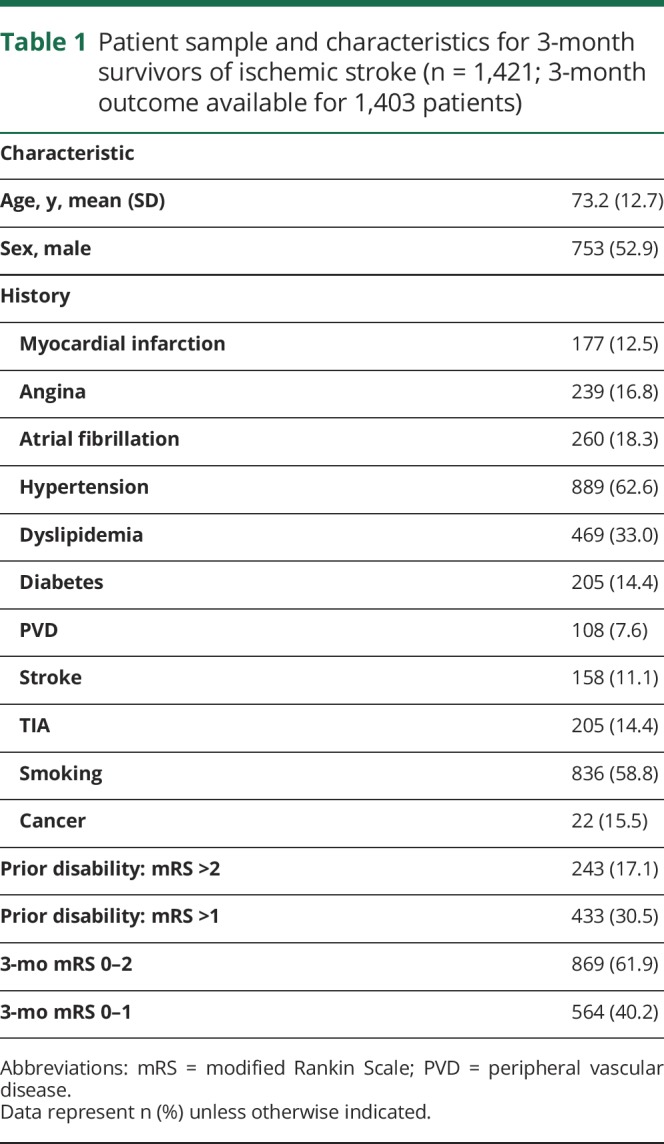
Patient sample and characteristics for 3-month survivors of ischemic stroke (n = 1,421; 3-month outcome available for 1,403 patients)

**Figure 2:**
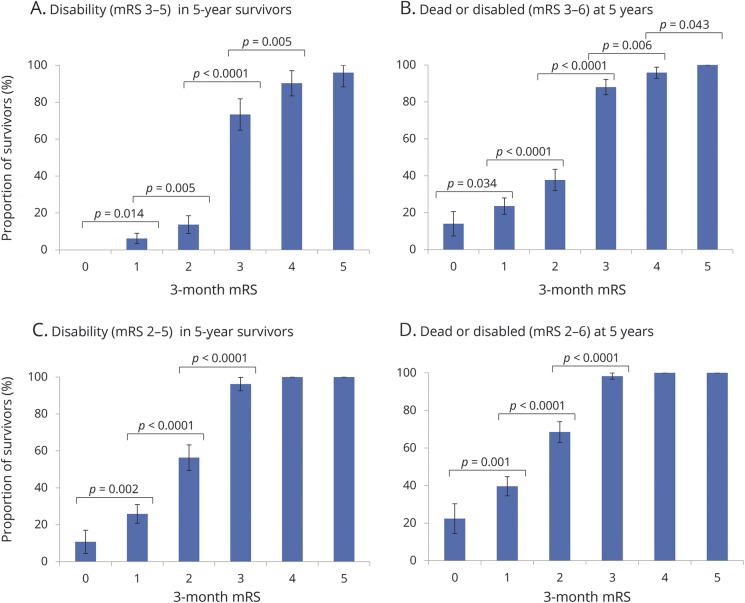
Five-year disability and death/disability outcomes in survivors of ischemic stroke, stratified by 3-month mRS scores The graphs show the proportion of 3-month survivors, also alive at 5 years, who were disabled at 5 years (A–C), and the proportion of 3-month survivors who were dead/disabled at 5 years (B–D), with disability defined as 5-year mRS score >2 (A and B) or mRS score >1 (C and D). Significant differences between mRS grades are indicated using *p* values from χ^2^ analysis. Bars represent 95% confidence intervals. mRS = modified Rankin Scale.

At 5 years, 465 (32.6%) 3-month survivors were dead and 214 (27.3%) of those alive had a 5-year mRS score >2 while 395 (50.3%) had an mRS score >1. One hundred seventy-three patients (12.1%) had not yet reached 5-year follow-up, but 1-year follow-up data were available for 169 of these, including everyone with a 3-month mRS. When disability was defined as mRS score >2, the proportion of survivors who were disabled at 5 years increased with 3-month mRS, with a bigger step change from mRS 2 to 3 vs mRS 1 to 2 (figure 2, A). Similar trends were observed with the composite outcome of 5-year death/disability (figure 2, B). On age- and sex-adjusted logistic regression, 3-month mRS was strongly related to 5-year disability (data available from Dryad, tables e-3 and e-4, doi.org/10.5061/dryad.609bp7m) and 5-year death/disability (data available from Dryad, tables e-5 and e-6), with AUC ≥0.8 for all 3 models. Both when including/excluding patients with premorbid mRS score >2, the AUCs for the ordinal model were consistently superior to the 0–2/3–5 and 0–1/2–5 dichotomies (e.g., for 5-year death/disability in all, ΔAUC [vs age/sex] = 0.132 for ordinal vs 0.124 for 0–2/3–5, *p* = 0.016; 0.080 for 0–1/2–5, *p* < 0.0001; ROC curves data available from Dryad, figures e-1 and e-2). When disability was defined as mRS score >1 (figure 2, C and D, and data available from Dryad, tables e-7 to e-10), the 2 dichotomies became similar in their relationship to 5-year disability and death/disability but the ordinal model remained superior when including/excluding those with premorbid mRS score >1 (ROC curves data available from Dryad, figures e-3 and e-4); e.g., for 5-year death/disability in all, ΔAUC = 0.071 for 0–2/3–5 vs 0.079 for 0–1/2–5, *p* = 0.22; 0.102 for ordinal, *p* = 0.0001.

**Figure 3:**
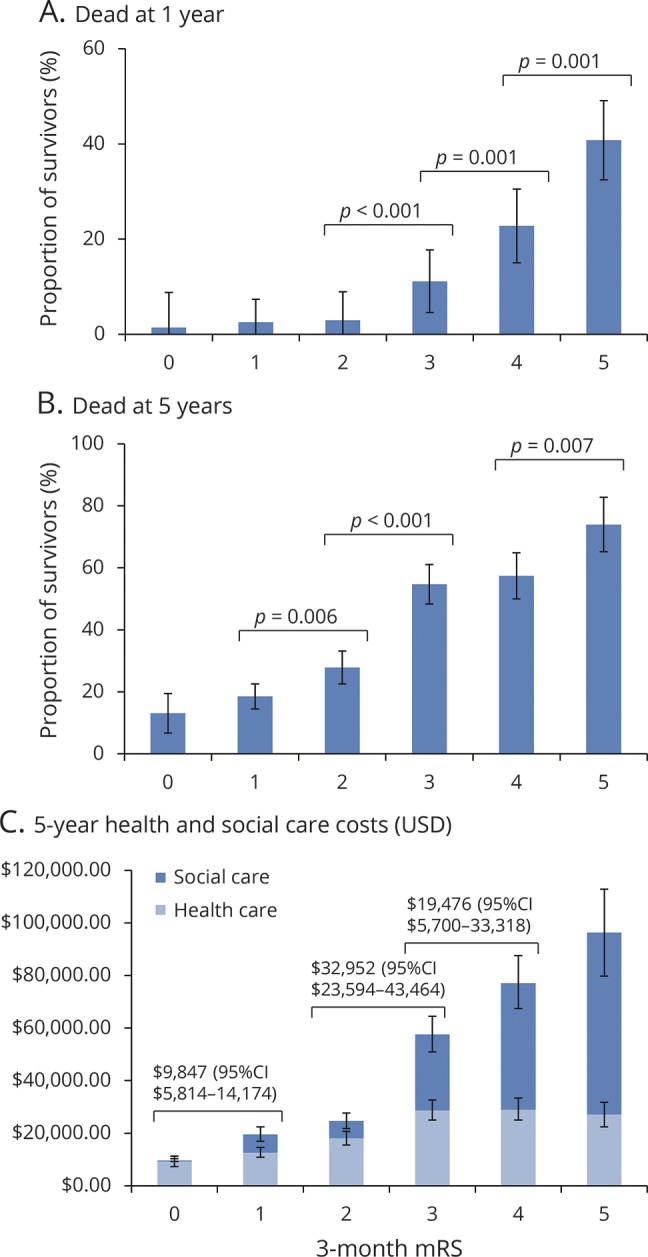
Deaths and health and social care costs in survivors of ischemic stroke, stratified by 3-month mRS scores The graphs show the proportion of 3-month survivors with full 5 years of follow-up who were dead at 1 year (A), dead at 5 years (B), and the cumulative 5-year health care costs for all survivors (C). Significant differences between mRS grades are indicated using *p* values from χ^2^ analysis (A and B) and mean cost differences with 95% CIs (C). CI = confidence interval; mRS = modified Rankin Scale; USD = US dollars.

Three-month mRS score was strongly related to 1-year mortality (figure 3, A), with 22 of 869 patients (2.5%) with 3-month mRS score of 0–2 dead at 1 year (no significant difference among 0, 1, or 2) vs 28 of 251 (11.2%) with mRS score 3, 41 of 180 (22.8%) with mRS score 4, and 42 of 103 (40.8%) with mRS score 5 (*p* ≤ 0.001 for each comparison). Consequently, on age- and sex-adjusted logistic regression, the 0–2/3–5 dichotomy better related to 1-year death than 0–1/2–5 while the ordinal scale was superior to both (table 2). On examining 5-year risk of death, mortality differences emerged between mRS 1 (66/356, 18.5%) and 2 (76/273, 27.8%, *p* = 0.006) while mRS 3 and 4 were similar (figure 3, B). The ordinal model remained the best fit for 5-year death (table 3, ROC curves data available from Dryad (figure e-5, doi.org/10.5061/dryad.609bp7m) while the 0–2/3–5 dichotomy performed better than 0–1/2–5 (*p* = 0.03). In particular, jumps in odds of 5-year death were seen from mRS 2 to 3 and 3–4 to 5. When patients with 3-month mRS of 6 were included in the regressions for 1- and 5-year death, the ordinal model remained superior to both dichotomies (data available from Dryad, tables e-11 and e-12).

**Table 2:**
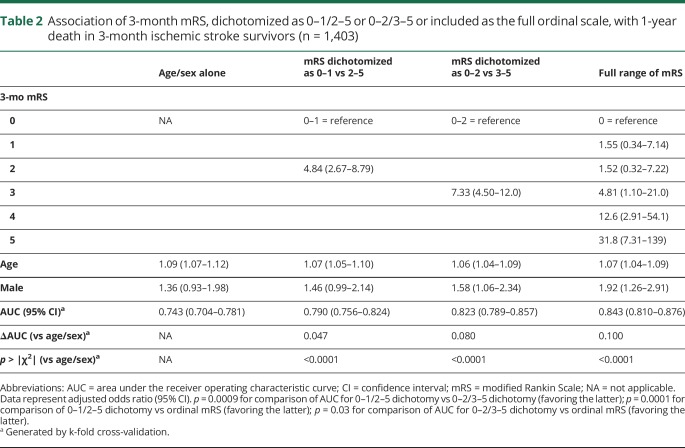
Association of 3-month mRS, dichotomized as 0–1/2–5 or 0–2/3–5 or included as the full ordinal scale, with 1-year death in 3-month ischemic stroke survivors (n = 1,403)

**Table 3:**
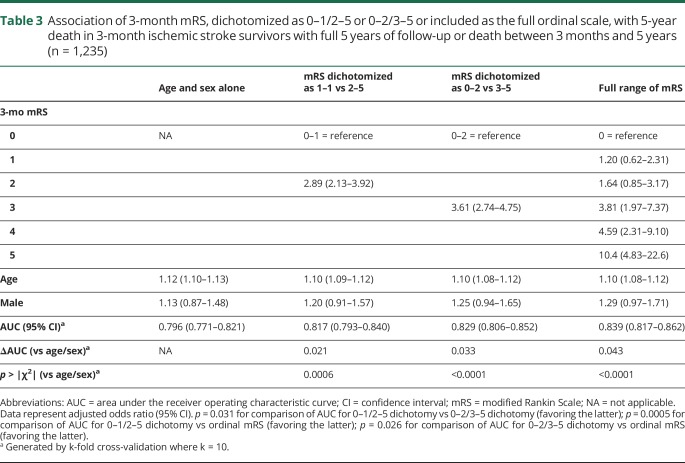
Association of 3-month mRS, dichotomized as 0–1/2–5 or 0–2/3–5 or included as the full ordinal scale, with 5-year death in 3-month ischemic stroke survivors with full 5 years of follow-up or death between 3 months and 5 years (n = 1,235)

When patients with premorbid mRS score >2 were excluded (data available from Dryad, tables e-13 and e-14, doi.org/10.5061/dryad.609bp7m), the ordinal model no longer significantly outperformed the 0–2/3–5 dichotomy but was still consistently superior to 0–1/2–5 (e.g., for 5-year death, ∆AUC = 0.037 for ordinal vs 0.026 for 0–2/3–5, *p* = 0.05; 0.019 for 0–1/2–5, *p* = 0.009; ROC curves data available from Dryad, figure e-6). However, the 0–2/3–5 dichotomy no longer had a significantly better fit than 0–1/2–5 for 1-year deaths (∆AUC = 0.072 for 0–2/3–5 vs 0.046 for 0–1/2–5, *p* = 0.086) and 5-year deaths (*p* = 0.30). When we further excluded those with premorbid mRS score >1 (data available from Dryad, tables e-15 and e-16), the 0–1/2–5 dichotomy became less useful with an AUC similar to age/sex alone for 1-year death (ΔAUC = 0.027, *p* = 0.17). The ordinal scale remained consistently superior, whereas the 0–2/3–5 dichotomy again was superior to 0–1/2–5 only in relation to 1-year death (ROC curves data available from Dryad, figure e-7).

Three-month mRS was also correlated with 5-year health and social care costs, which increased with each mRS grade (figure 3, C). Patients with 3-month mRS score of 5 accrued the highest costs despite high mortality, and there were significant differences in costs between patients with 3-month mRS score of 1 vs 2, 2 vs 3, and 3 vs 4 (e.g., mean difference between mRS 2 and 3: $32,952, 95% confidence interval $23,594–$43,464) vs the insignificant difference between mRS 1 and 2 ($5,104, −$1,090 to $10,614). Five-year costs were best captured by the ordinal model, which had the lowest MAE, i.e., minimized error (table 4). Results were similar when censored cases were included (data available from Dryad, table e-17, doi.org/10.5061/dryad.609bp7m); even upon excluding patients with premorbid mRS >2 and mRS >1 (data available from Dryad, tables e-18 and e-19), ordinal analysis was superior and 0–1/2–5 inferior to the 0–2/3–5 dichotomy.

**Table 4:**
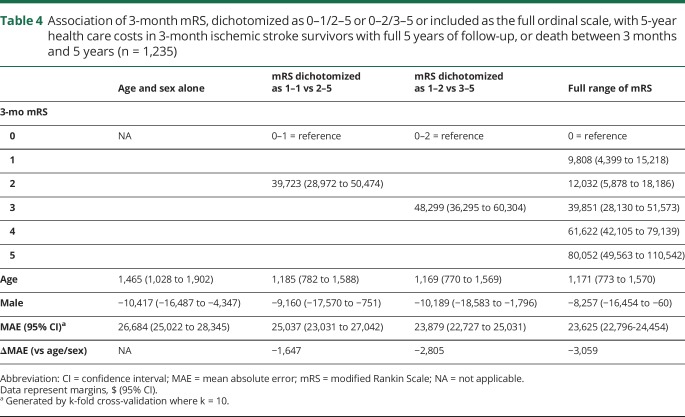
Association of 3-month mRS, dichotomized as 0–1/2–5 or 0–2/3–5 or included as the full ordinal scale, with 5-year health care costs in 3-month ischemic stroke survivors with full 5 years of follow-up, or death between 3 months and 5 years (n = 1,235)

## Discussion

We have demonstrated that the ordinal mRS relates better to 5-year disability (whether defined as mRS >1 or >2), death/disability, mortality, and health/social care costs, than both the 0–1/2–6 and 0–2/3–6 dichotomies. In addition to further validating the mRS as an outcome measure, these findings have implications for trials using the mRS as the primary outcome measure.

First, our findings favor retaining the full ordinal range of the mRS over dichotomizing it for analysis, by adding clinical data to prior arguments about statistical efficiency. Efficiency depends on expected treatment effects and does not always favor ordinal analysis.^9^ However, a clinically robust primary outcome measure should capture differences in long-term disability, care needs (costs), and survival—objective treatment goals in neurologic disease—and our analyses demonstrate that the ordinal mRS best reflects these outcomes, with differences seen across the range of the scale. These results imply that any shifts in mRS can be meaningful, and that it may be misleading to consider certain states as being equivalent with respect to long-term outcomes. It is also inappropriate to view patients with premorbid disability as having no potential to benefit from disability-lowering treatments, given significant differences in long-term outcome among patients with 3-month mRS grades 3, 4, and 5, and certainly between mRS 2 and 3.

Second, our results highlight the substantial proportion of patients with premorbid disability who risk exclusion with dichotomous approaches. The 0–1/2–6 dichotomy would have excluded 30.5% of our cohort from attaining a favorable outcome even prestroke and was more likely to exclude older, female, and socioeconomically deprived patients. Defining a favorable outcome as mRS score 0–2 is less exclusionary, but would still have prevented 17.2% of our patients from demonstrating any treatment benefit. Previous inpatient studies have reported higher rates of premorbid disability.^25^ Even when trials use block randomization or actively enroll older patients, such as the Third International Stroke Trial, their sample will still be unrepresentative if they exclude patients with premorbid disability, as did the Third International Stroke Trial.^26^ Ageism has been noted in interventional stroke studies,^27^ and older patients, women, and socioeconomically deprived patients are less likely to receive appropriate acute stroke care.^28,29^ By permitting patients with premorbid disability to demonstrate treatment benefit, ordinal analysis can encourage trials to enroll such patients and better represent the stroke population. However, clinical trials have generally enrolled healthier patients to optimize the detection of treatment effects, and enrolling patients with various levels of premorbid disability can complicate sample-size calculations and add another factor for adjustment when comparing treatment and control arms.

Third, for acute stroke trials that still wish to dichotomize the mRS, our data suggest that in addition to promoting exclusion of more patients, trials using the 0–1/2–6 dichotomy risk ultimately comparing more similar patient groups with respect to long-term outcomes than those using a 0–2/3–6 dichotomy, since patients with a 3-month mRS score of 2 seem likely to fare similarly to those with an mRS of 1—and much more favorably than those with mRS of 3 (“step change”), considering long-term outcomes. This is a concern given the 0–1/2–6 dichotomy's popularity in recent trials.^3,4^ However, 0–2/3–6 may not always be the most optimal dichotomy for trials based on other factors such as expected case mix and stroke severity. For example, trials of hemicraniectomy in malignant middle cerebral artery infarction used a dichotomy of 0–4/5–6.^30^

Fourth, our findings demonstrate nonlinear trends in the long-term outcomes predicted by different mRS grades, suggesting that not all shifts should be weighted equally in an ideal ordinal analysis. The shift from 0–2 to 3–6 appears to merit the highest weighting, but there are also jumps in mortality and care costs from 3 to 4 to 5. This is in contrast to conventional approaches to ordinal analysis, such as proportional odds logistic regression, which assume that all step increases in the mRS are equal. The utility-weighted mRS may be one method to address this issue, although it most values transitions from 3 to 4 to 5.^31^ However, as recently suggested in a simulation study, differently weighted approaches such as the utility-weighted mRS may reduce the statistical power of randomized trials vs conventional ordinal analysis, although in this case, it was still more efficient than dichotomous approaches.^32^ Using a sliding dichotomy, in which a good outcome—and thereby the state transition of interest for a given patient—is defined based on initial severity or other characteristics is more informative than a fixed dichotomy, but still fails to utilize information from the entire range of mRS outcomes.^5^

Our analysis has several strengths, including a robust population-based design, high rates of ascertainment of all incident strokes, completeness of follow-up, replication of findings for several outcomes, and generalizability, with similar 5-year mortality and disability rates as prior population-based studies.^33,34^ However, there are some potential shortcomings. First, a randomized-controlled trial is required to prove a causal association between shifting patients to lower mRS scores and reducing long-term disability, mortality, and costs, and to validate the differential value of various state transitions suggested by our analyses. Our findings would also benefit from further validation in other large cohort studies. Second, since assessors were not blinded to prior mRS scores, 1- and 5-year disability assessments could have been unfavorably biased in patients with higher 3-month mRS scores. However, ordinal analysis remained superior with different disability definitions, and trends were similar for 5-year death and costs, which are untainted by disability assessments. Third, we only studied patients with ischemic stroke; distinctions among mRS grades may differ in other diseases. Fourth, given that our aim was to examine how the mRS relates to long-term outcomes, we did not adjust for all the potential factors that could also influence long-term disability, mortality, and costs, including stroke subtype, impairment on the NIH Stroke Scale, various cardiovascular and noncardiovascular comorbidities (such as cancer), recurrent strokes, socioeconomic status, or adherence to rehabilitation or secondary prevention. Ethnicity, potentially a relevant factor for such long-term outcomes, was also not examined; the Oxford Vascular Study population is 95% white. Such factors may need to be accounted for by studies seeking to more accurately estimate long-term outcomes for purposes like resource allocation. Fifth, given our wide recruitment period, secular trends in society and the health care system may have shifted the mRS distribution of our population over time. For instance, increasing longevity could have resulted in a higher proportion of patients with increased premorbid mRS over time; therefore, our estimate of the burden of premorbid disability may be an underestimate compared with present-day populations.

Furthermore, examining relationship to long-term outcomes such as mortality and costs is only one way to compare different 3-month mRS representations. Other measures that have been previously related to 3-month mRS include simultaneously assessed metrics such as quality of life (concurrent validity),^35^ or retrospective metrics such as home time within the first 90 days after hospitalization.^36^ Such metrics could also be used to examine differences between the different 3-month mRS representations in future studies, and it is possible that their findings may differ from ours.

Our findings demonstrate that ordinal analysis of the 3-month mRS better relates to long-term outcomes of ischemic stroke than either dichotomy. Exclusion of patients with higher premorbid disability by use of dichotomous primary outcomes will also result in unrepresentative samples. Similar considerations may apply to other comparable ordinal scales used for trial outcomes.

## Publication history

Received by *Neurology* March 21, 2018. Accepted in final form August 8, 2018.

## Glossary

AUC: area under the receiver operating characteristic curve
GLM: generalized linear model
GP: general practitioner
MAE: mean absolute error
mRS: modified Rankin Scale
ROC: receiver operating characteristic
